# Platelet-rich plasma ameliorates lipopolysaccharide-induced cardiac injury by inflammation and ferroptosis regulation

**DOI:** 10.3389/fphar.2022.1026641

**Published:** 2022-10-18

**Authors:** Yuheng Jiao, Qingyu Zhang, Jiayan Zhang, Yafang Zha, Jian Wang, Yanyan Li, Song Zhang

**Affiliations:** ^1^ Department of Cardiology, Renji Hospital, Shanghai Jiao Tong University School of Medicine, Shanghai, China; ^2^ Hospital of Stomatology, Jilin University, Changchun, China; ^3^ Department of Cardiology, Xinhua Hospital, Shanghai Jiao Tong University School of Medicine, Shanghai, China

**Keywords:** platelet-rich plasma, LPS, cardiac injury, inflammation, ferroptosis

## Abstract

Sepsis-induced myocardial dysfunction (SIMD) is a fatal disease with no specific treatment worldwide to this day. As a biological product, platelet-rich plasma (PRP) has attracted much attention due to its diverse and potential biological effects. However, its role in lipopolysaccharide (LPS)-induced cardiac injury has not been fully investigated. This study aimed to explore the mechanism of PRP in SIMD. PRP (30 µL) was injected *in situ* into the heart, and LPS (10 mg/kg) was injected intraperitoneally into mice. Neonatal rat cardiomyocytes were treated with LPS (1 μg/ml) for 24 h. The results showed that, compared with the LPS group, PRP significantly decreased the levels of Lactate dehydrogenase (LDH) and Creatine Kinase MB (CK-MB), and improved cardiac function. In addition, PRP markedly decreased the Malonic dialdehyde (MDA) content, and increased the Superoxide dismutase (SOD) activity and Glutathione (GSH) level, demonstrating that PRP alleviated LPS-induced oxidative stress. The Western blot and qPCR results showed that LPS-induced ferroptosis and inflammation effects *in vivo* and *in vitro* were ameliorated after PRP treatment. Moreover, PRP can alleviate erastin-induced ferroptosis and improve cell viability. Mechanistically, p-AKT and p-mTOR expressions were down-regulated after treatment with LPS, while PRP pretreatment could reverse this effect. In summary, our study demonstrated that PRP could play a unique role in reducing LPS-induced cardiac injury through regulation of AKT/mTOR signaling pathways. These findings provide a new therapeutic direction for treating SIMD.

## 1 Introduction

Sepsis is a common systemic disease with high mortality worldwide (Singer et al., 2016). It has been reported that sepsis can infect multiple organs throughout the body, such as the kidney, lung, and heart, causing severe damage (Cheng et al., 2017; Sun et al., 2018; Yoo et al., 2020). As the blood supply center of the body, the heart plays a vital role in life, and sepsis seriously affects the systolic function of the heart, resulting in failure of the cardiac output to meet the needs of metabolism, which leads to a series of symptoms ad complications (Arfaras-Melainis et al., 2020; Luo et al., 2020; Tan et al., 2021). Previous studies have shown that inflammation is an important part of the pathogenesis of sepsis-induced myocardial dysfunction (SIMD) (Li N. et al., 2019; Zhao et al., 2020; Dai et al., 2021).

Although many mechanisms are involved in the development of septic cardiomyopathy, such as endoplasmic reticulum stress, mitochondrial dysfunction, apoptosis, and oxidative stress, inflammation remains the focus of research (Li et al., 2018; Pang et al., 2019). In patients with septic cardiomyopathy, various proinflammatory factors, such as IL-6, IL-1β, and TNF-α, are significantly increased, leading to cascade reactions (Koyani et al., 2020). A recent study has found that ferroptosis also plays an essential role in this mechanism (Li N. et al., 2020). Current evidence demonstrates that in addition to cell death forms such as apoptosis, autophagy, and necrosis, ferroptosis is a crucial intervention target for septic cardiomyopathy (Shan et al., 2021). Ferroptosis is a controllable cell death mode characterized by the accumulation of iron-dependent lipid peroxides, which are involved in various physiological and pathological processes and can play a role in the development and treatment of certain diseases after being regulated.

Platelet-rich plasma (PRP) is a new platelet concentrate prepared from peripheral blood by centrifugation and contains about 3–6 times the whole blood concentration (Ornetti et al., 2016). PRP has been widely used in many fields such as stomatology, fracture healing, and neurology due to its simple preparation and abundant sources (Ozsagir et al., 2020; Zhu et al., 2020; Al-Hamed et al., 2021). PRP contains not only high concentrations of platelets but also many growth factors, including EGF, VEGF, TGF-β, PDGF, etc., (Masuki et al., 2016). Many growth factors and cytokines have anti-inflammation and anti-apoptosis biological functions (Zheng et al., 2016; Tsai et al., 2018); they play a vital role in improving and maintaining micro-environment homeostasis and the regeneration and repair of blood vessels and tissues (Al-Hamed et al., 2019). However, at present, there are relatively few studies on the role of PRP in cardiology, especially in SIMD.

This study confirmed that PRP could play a protective role in septic cardiomyopathy. Our results manifested that PRP could improve cardiac function and reduce the mortality of septic cardiomyopathy by inflammation and ferroptosis regulation *in vitro* and *in vivo*.

## 2 Materials and methods

### 2.1 Animals and treatment

All C57BL/6 mice (6–8w, 20–25 g) were purchased from Slaccas Laboratory Animal Breeding Co., Ltd. (Shanghai, China). All animals were raised in SPF environment and given sufficient food and water, with a light and dark cycle of 12 h. After a week of adaptation, mice were randomly divided into three groups: control group, Lipopolysaccharide (LPS) group, and Lipopolysaccharide + Platelet-rich plasma (LPS + PRP) group. After continuous inhalation of isoflurane, the mice were anesthetized and the skin was cut at the strongest apical beat. The pectoralis major and pectoralis minor muscles were separated, and then the heart was squeezed out. At last, PRP or PBS (30 µL) was injected into 3-4 locations of the left ventricular wall. Four days later, the mice were injected with LPS (10 mg/kg) by intraperitoneal injection for 12 h. To monitor the survival rate, we injected 30 µL PRP or PBS into the mice *in situ*, and then injected 15 mg/kg LPS intraperitoneally to observe the survival rate for 48 h.

### 2.2 Platelet-rich plasma preparation

PRP was prepared by double-spin centrifugation protocol (Zhao et al., 2021). First, the blood was collected from a Sprague-Dawley rat heart with special tubes, which contain anticoagulants, and immediately centrifuged at 200 g for 10 min. This short step divides the blood into three layers, and the red blood cells in the bottom layer were discarded. Next, the components in the middle and upper layer were transferred to another centrifuge tube. After centrifugation at 400 g for 10 min, 50% of the upper supernatant was carefully removed, and the remainder was filtered with a 0.22 mm filter. Importantly, we need to calculate the platelet content and compare it with the number of blood. Finally, the liquid was stored in a −80°C, refrigerator.

### 2.3 Cardiac function measurement

Cardiac ultrasound was performed after LPS treatment for 12 h. Briefly, the hair in the chest of mice was removed with a depilating agent. After continuous inhalation of isoflurane, the mice was placed on a heating pad to maintain the body temperature of 37°. Two-dimensional guided M-mode measurements of left ventricular (LV) diameter were obtained from short-axis views at the papillary muscle level for at least three cardiac cycles by ultrasound instrument (Vivid 7; GE Medical, Milwaukee, WI, United States). The left ventricular ejection fraction (LVEF) and fractional shortening (FS) were calculated using computer algorithms.

### 2.4 Biochemical and serum analyses

According to the instructions, the contents of LDH(Huili Biotec, C018), CK-MB(Huili Biotec,C060), SOD (Jiancheng Biotec, A001-1), MDA (Jiancheng Biotec, A003-1), GSH(Jiancheng Biotec, A006-2), and non-heme iron (Huili Biotec, C016) in serum were detected using different kits, as well as heart tissues. Whole blood samples were placed at room temperature for 2 h or overnight at 4°C, then centrifuged at 2°C–8°C 3000 rpm for 15 min, and the supernatant was taken for subsequent detection. Accurately weigh the weight of animal tissue by weight (mg): volume (ul) = 1 : 9 ratio of 9 times the volume of the homogenate medium, under the condition of ice water bath, mechanical homogenate, prepared into 10% homogenate, 2,500–3,000 rpm, centrifuged for 10 min, the supernatant was measured. The relevant operation according to the kit instructions.

### 2.5 Detection of IL-6, IL-1β, and TNF-α

IL-6 (Invitrogen, 88–7013), IL-1β (Invitrogen, 88–7064), and TNF-α (Invitrogen, 88–7324) levels of serum were analyzed using different ELISA Kits according to the instructions. Besides, IL-6 (Multi Science, EK306/3-01), IL-1β (Multi Science, EK301B/3-02), and TNF-α (Invitrogen, 88–7340) levels of cell culture medium were detected using the same method. Briefly, the sample and the standard were added to the test hole respectively, and then the working solution was added and incubated in a constant temperature incubator for 60 min. After the incubation, the wells were repeatedly washed 3-4 times with wash buffer, each hole were added to the color reagent and incubated in the dark for 15min, and finally added termination solution. The absorbance of each well was measured at 450 nm and the concentration of the corresponding sample was calculated according to the standard curve.

### 2.6 Cell treatment

**FIGURE 1:**
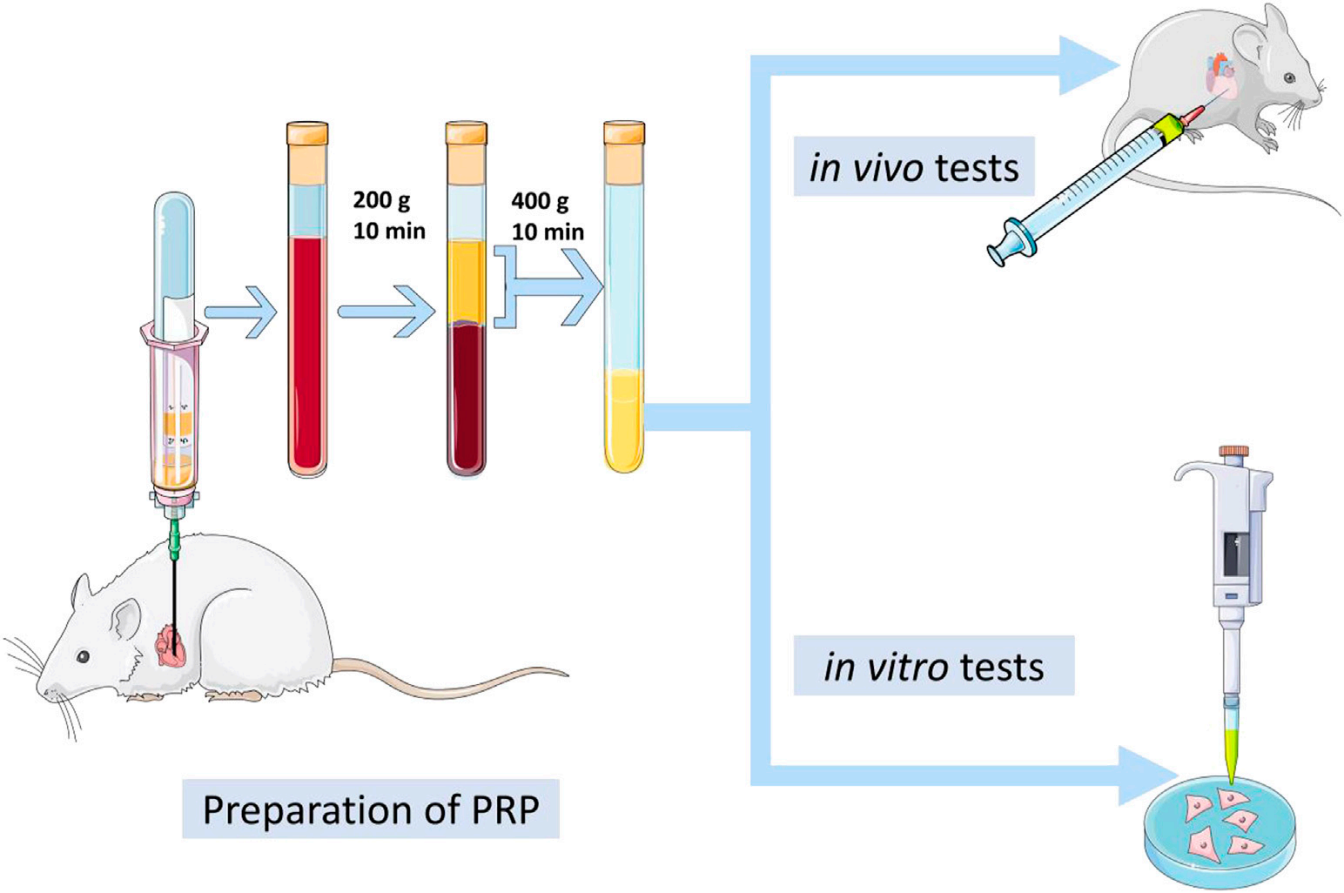
The flow diagram for the experimental procedures.

Neonatal rat cardiomyocytes (NRCM) were obtained according to the previous method (Zhang et al., 2018). Different concentrations of PRP (1%,5%, and 10%) and inhibitors (AKTi and Rapamycin, MCE, HY-10355, HY-10219) were added into the medium containing 10% fetal bovine serum (Sigma, F8318) and 1% penicillin-streptomycin solution (Hyclone, SV30010), and the same amount of PBS was added to the control group. After 2 h of incubation in a 37°C, incubator, LPS (1 μmol/L) was added for 24 h (Figure1).

### 2.7 Western blot

According to the previous method, we extracted proteins from cells and heart tissues for Western blot experiments (Jiao et al., 2022). In short, we use 7.5–12.5% SDS-PAGE gel for protein separation and transfer it to PVDF membranes. After blocking with 5% nonfat dry milk at room temperature for at least 1 h, the membranes were washed three times with TBST and incubated with primary antibodies overnight in a 4°C refrigerator. The corresponding primary antibodies are as follows: GAPDH (Abcam, ab181602), PTGS2 (Abclonal, A1253), AKT (Proteintech, 10176-2-AP), Phospho-Akt (Abclonal, AP1259), Phospho-mTOR (CST, 5536T), mTOR (CST, 2983T). After collecting the primary antibody, the membranes were reacted with HRP-conjugated secondary antibodies (Beyotime, A0208) for 1 h. At last, the immune blots were observed by enhanced chemiluminescence ECL (Epizyme, SQ201).

### 2.8 Real-time PCR analysis

According to the instructions, we use Trizol to isolate total RNA from cells and tissues. Prime-ScriptTMRT reagent kit (Yeasen, 11141ES60) was used to reverse total RNA into cDNA. Amplification and quantitative PCR detection were performed using SYBR Green (Yeasen, 11202ES08). Normalization of target gene expression to GAPDH mRNA content. The primer sequences were as shown in Table 1.

**TABLE 1:** Table of primers sequences for Real-Time PCR.

Gene symbol	Forward primer	Reverse primer
GAPDH (Mus musculus)	GGC​AAA​TTC​AAC​GGC​ACA​GTC​AAG	TCG​CTC​CTG​GAA​GAT​GGT​GAT​GG
IL-6 (Mus musculus)	CTC​CCA​ACA​GAC​CTG​TCT​ATA​C	CTC​CCA​ACA​GAC​CTG​TCT​ATA​C
IL-1β (Mus musculus)	CAC​TAC​AGG​CTC​CGA​GAT​GAA​CAA​C	TGT​CGT​TGC​TTG​GTT​CTC​CTT​GTA​C
TNF-α (Mus musculus)	ATG​TCT​CAG​CCT​CTT​CTC​ATT​C	ATG​TCT​CAG​CCT​CTT​CTC​ATT​C
PTGS2 (Mus musculus)	CTG​GTG​CCT​GGT​CTG​ATG​ATG​TAT​G	GGA​TGC​TCC​TGC​TTG​AGT​ATG​TCG
GAPDH (Rattus norvegicus)	AGT​TCA​ACG​GCA​CAG​TCA​AGG​C	CGA​CAT​ACT​CAG​CAC​CAG​CAT​CAC
IL-6 (Rattus norvegicus)	AGT​TGC​CTT​CTT​GGG​ACT​GAT​GTT​G	AGT​TGC​CTT​CTT​GGG​ACT​GAT​GTT​G
IL-1β (Rattus norvegicus)	AAT​CTC​ACA​GCA​GCA​TCT​CGA​CAA​G	TCC​ACG​GGC​AAG​ACA​TAG​GTA​GC
TNF-α (Rattus norvegicus)	CCA​CGC​TCT​TCT​GTC​TAC​TGA​ACT​TC	AGA​TGA​TCT​GAG​TGT​GAG​GGT​CTG​G
PTGS2 (Rattus norvegicus)	CAC​ATT​TGA​TTG​ACA​GCC​CAC​CAA​C	AGT​CAT​CAG​CCA​CAG​GAG​GAA​GG

### 2.9 Measurement of ROS in cell

After cells were treated with LPS and PRP, the medium was discarded and washed with PBS three times. According to the instructions, DCFH-DA (Beyotime, S0033S) was added to the medium and cultured for 30 min. Finally, the cells were washed with PBS three times, and the brightness was observed by fluorescence microscope (Olympus, Tokyo, Japan).

### 2.10 Cell viability assay

According to the instructions, we used cell count kit-8 (CCK-8, Beyotime, C0041) to detect cell viability. First, 5 × 10^3^ cells were seeded in a 96-well plate and cultured for 24 h. Erastin (Abmole, M2679,5 µM) and PRP were added to cells for 24 h. After that, CCK8 (10 µL) was added to each well, and the plate was incubated for 3 h at 37°C. Finally, the absorbance at 450 nm was detected.

### 2.11 Histological analysis

After treated with PRP and LPS, mice were anesthetized with isoflurane and the heart tissues were taken out. According to the previous experiments, we performed immunofluorescence to detect the content of ROS in tissues and Hematoxylin and eosin (H&E) staining (Jiao et al., 2022). Briefly, fresh heart tissues were fixed in 4% paraformaldehyde and embedded in paraffin. Hematoxylin and eosin (H&E, Beyotime, C0105S) solution was used for staining analysis. The heart tissues were made into frozen sections and incubated with ROS staining solution, and then washed with PBS and incubated with DAPI staining solution.

### 2.12 Prussian blue staining

The heart sections were dewaxed and hydrated, then transferred to working solution for incubation at room temperature for 20 min, and washed with distilled water at the end of incubation. Finally, the sections were observed under a light microscope (Song et al., 2021).

### 2.13 Data analysis

All values are shown as means ± standard deviation (SD). Differences between groups were analyzed by an unpaired, two-tailed Student t-test (two groups) or ANOVA (three or more groups) followed by Bonferroni’s correction if needed. Graph Pad Prism 5.0 software was used for the statistical test, and *p* < 0.05 was regarded as statistically significant.

## 3 Results

### 3.1 The characters of platelet-rich plasma

The average number of platelets in PRP is 6,399 ± 2,334 × 10^9^/L, while the average number of platelets in whole blood is 887 ± 325 × 10^9^/L. Therefore, the number of platelets in PRP is about 7 times higher than in whole blood. The numbers of different blood cells are detailed in (Table 2).

**TABLE 2:** Complete blood count of whole blood and PRP.

Composition	Whole blood mean ± SD	PRP mean ± SD	*p* value
Platelets x 10^9^/L	887 ± 325	6,399 ± 2,334	<0.05
RBCs x 10^12^/L	7.2 ± 0.49	1.8 ± 0.62	<0.05
WBCs x 10^9^/L	5.25 ± 0.80	12.8 ± 4.07	<0.05

WBC, white blood cell; RBC, red blood cell.

### 3.2 Lipopolysaccharide activated inflammation and ferroptosis in the heart

**FIGURE 2:**
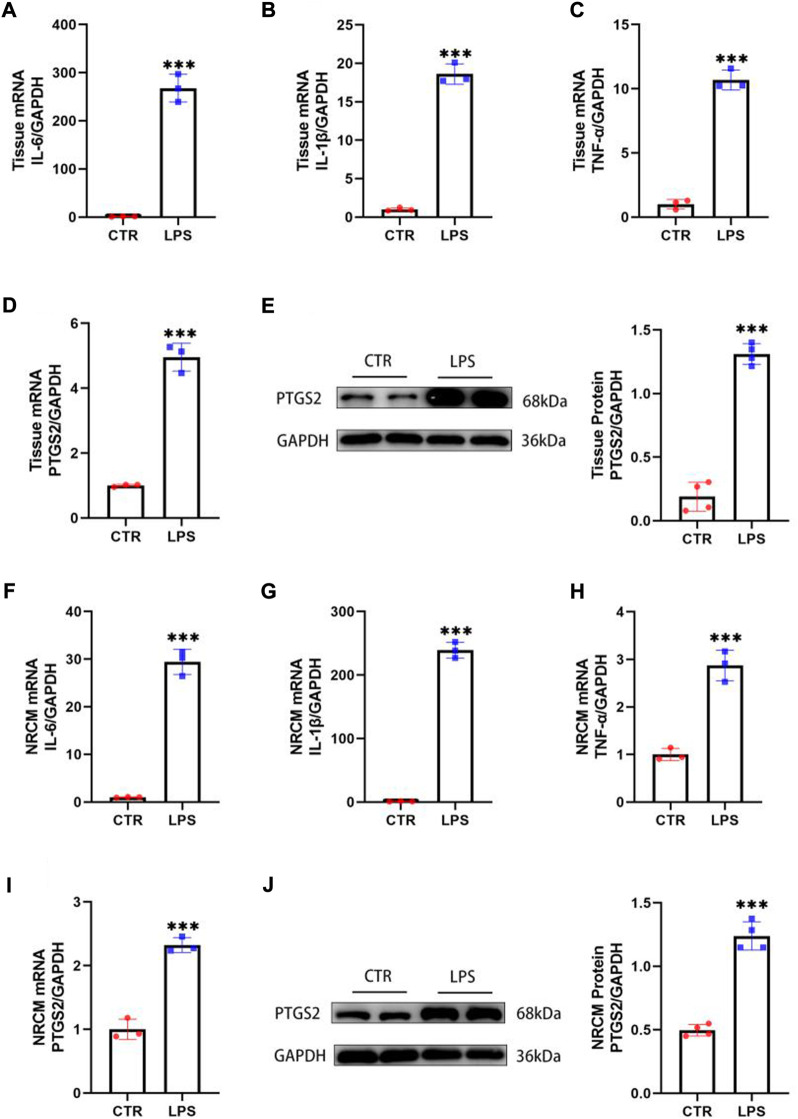
LPS activated inflammation and ferroptosis in the heart. **(A–D)** The mRNA expression levels of IL-6, IL-1β, TNF-α and PTGS2 in the hearts of mice treated with LPS (*n* = 3). **(E)** The protein levels of PTGS2 in tissues (*n* = 4). **(F–I)** The mRNA expression levels of IL-6, IL-1β, TNF-α and PTGS2 in NRCMs treated with LPS (*n* = 3). **(J)** The protein levels of PTGS2 in tissues (*n* = 4). *** indicates *p* < 0.001.

Inflammation has been considered a key pathway for LPS-induced cardiac injury. Notably, PTGS2 is one of the important indicators of ferroptosis, and its content is significantly increased in heart tissues stimulated by LPS, suggesting that ferroptosis could also be involved in the process of SIMD (Shen et al., 2007). To confirm the inflammation and ferroptosis pathways are involved in LPS-induced cardiac injury, we injected mice intraperitoneally with LPS (10 mg/kg), and after 12 h of treatment, the mRNA and proteins were extracted from the heart tissues. The qPCR results manifested that the expression levels of inflammatory cytokines such as IL-6, IL-1β, and TNF-α in the heart tissue of mice treated with LPS were markedly increased (Figures 2A–C), indicating that the inflammatory pathway was activated. In addition, the expression of PTGS2 was also increased compared with the control group (Figure 2D), suggesting that the ferroptosis process was also involved in SIMD. The Western blot results further confirmed the activation of ferroptosis pathways (Figure 2E). Moreover, these findings were also verified in neonatal rat cardiomyocytes (NRCMs), and the qPCR and Western blot results were consistent with the animal experiments (Figures 2F–J). Therefore, we speculated that the inhibition of inflammation and ferroptosis could alleviate the damage caused by LPS in cardiomyocytes.

### 3.3 The effect of platelet-rich plasma on lipopolysaccharide-induced cardiac injury

**FIGURE 3:**
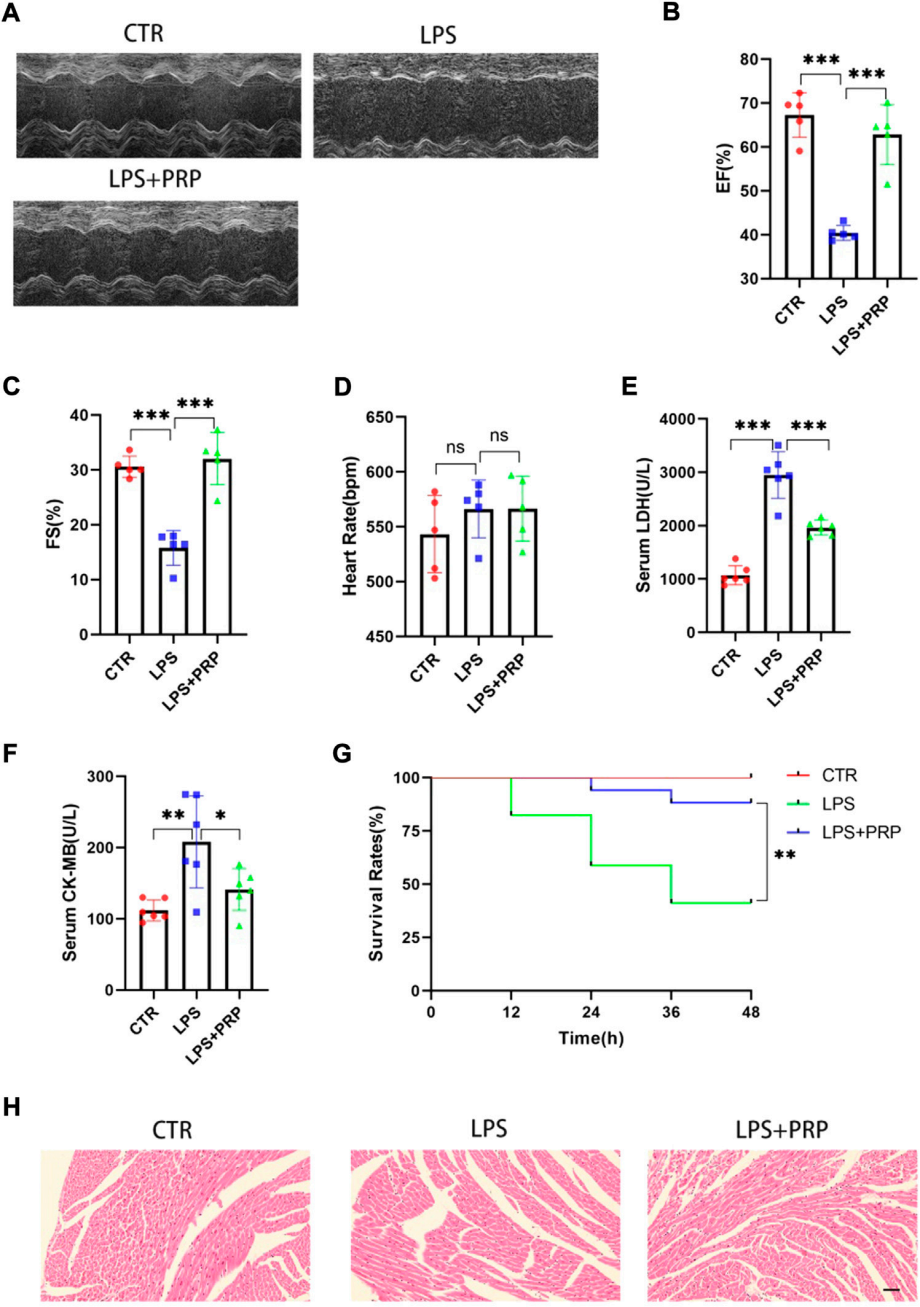
PRP alleviated LPS-mediated cardiac injury. **(A)** Representative images of M-mode echocardiogram. **(B–D)** The ejection fraction (EF), fractional shortening (FS), and heart rate of mice (*n* = 5). **(E)** and **(F)**The levels of LDH and CK-MB in serum (*n* = 6). **(G)** The survival rate of mice injected with PBS (30 µL), LPS (15 mg/kg), and LPS + PRP (30 µL) (*n* = 17 per group). **(H)** H&E staining in hearts compared with LPS and different groups (scale bar = 50 μm). * indicates *p* < 0.05. ** indicates *p* < 0.01. *** indicates *p* < 0.001. ns indicates *p* > 0.05.

Previous studies suggested that LPS could reduce the cardiac ejection fraction, hindering the cardiac blood supply from meeting the metabolism needs and resulting in corresponding symptoms or even death (Shan et al., 2021). However, studies have reported that PRP injected into the infarcted sheep myocardial tissue significantly promoted the formation of blood vessels in the infarcted area, which indicates that PRP has a protective effect on heart tissue (Gallo et al., 2013). To verify the role of PRP in SIMD, we used C57BL/6 mice for subsequent experiments and injected PRP (30 µL) into the mouse heart *in situ*. Four days later, mice were injected with LPS by intraperitoneal injection. After 12 h, the results of cardiac ultrasound showed that the LVEF and FS of mice injected with LPS intraperitoneally were significantly lower than those of the control group, while the cardiac function of mice treated with PRP in advance was improved compared with mice treated with LPS alone (Figures 3A–C). Regarding the heart rate, there was no significant difference between each group (Figure 3D). As one of the markers of heart injury, the change of LDH content has important indicative significance. Through the detection of serum, we found that LPS could significantly increase the LDH level, indicating that LPS could cause serious cardiac injury. In addition, the expression of CK-MB, another important marker of cardiac injury, was significantly elevated after LPS treatment. However, PRP can significantly reverse these results (Figures 3E,F). The survival rate of mice treated with LPS for 48 h was 41.2%, while that of mice treated with PRP was 88.2%, indicating that PRP could significantly reduce mortality (Figure 3G). H&E staining demonstrated that cardiomyocyte cross-sectional area did not change significantly among the three groups (Figure 3H).

### 3.4 Platelet-rich plasma attenuated lipopolysaccharide-induced oxidative stress and lipid peroxidation *in vivo*

**FIGURE 4:**
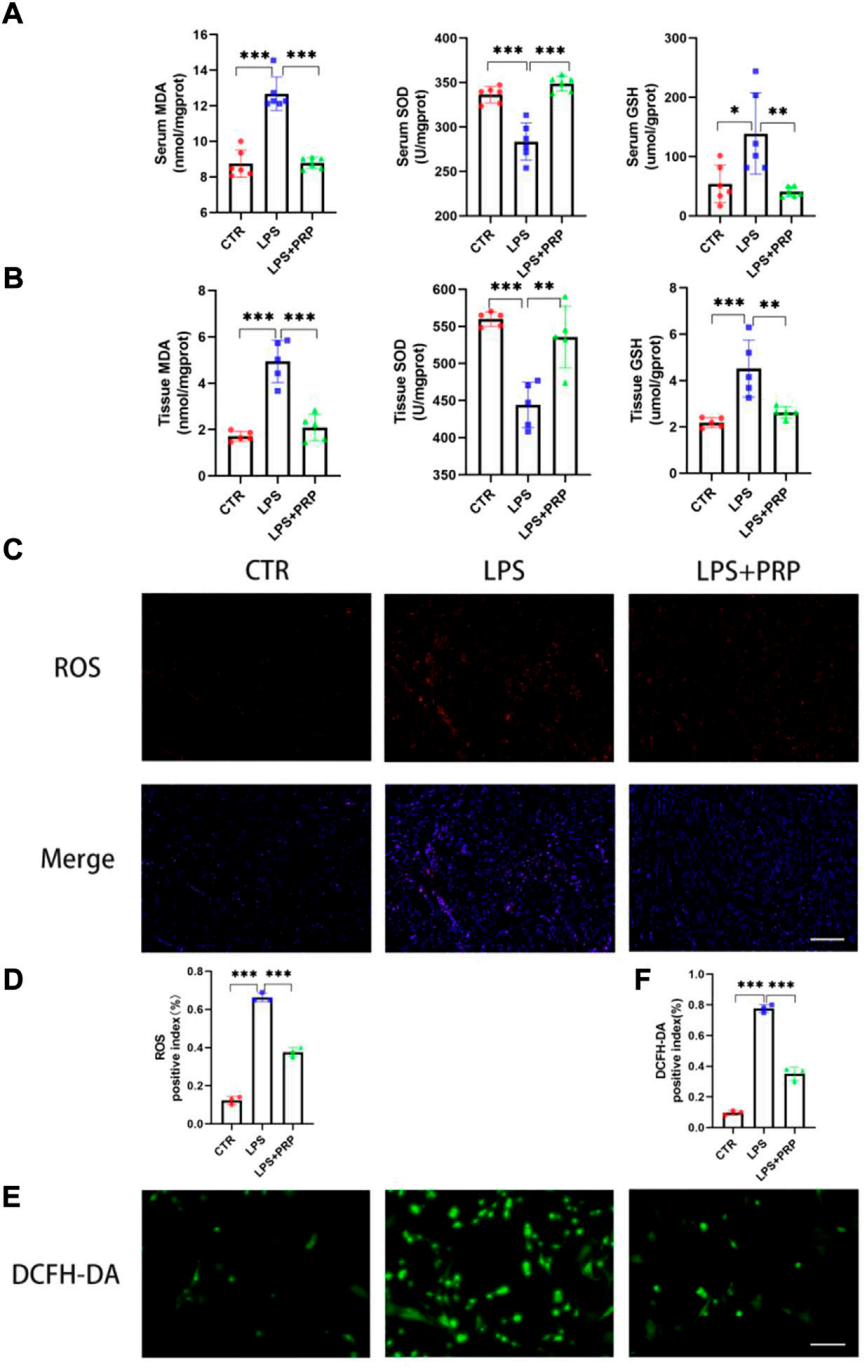
PRP attenuated LPS-induced oxidative stress and lipid peroxidation *in vivo*. **(A)** Effects of LPS on the levels of MDA, SOD, and GSH in serum (*n* = 6). **(B)** The levels of MDA, SOD, and GSH in tissues after LPS and PRP treatment (*n* = 5). **(C–D)** Effect of LPS on ROS levels in heart tissues (*n* = 3, scale bar = 20 µm). **(E)** and (**F)** Effect of LPS on cellular ROS levels in NRCMs (*n* = 3, scale bar = 20 µm). * indicates *p* < 0.05. ** indicates *p* < 0.01. *** indicates *p* < 0.001.

Free radicals act on lipid peroxidation, and the oxidation product is malondialdehyde (MDA), which affects mitochondrial respiratory chain complexes and key enzyme activities in mitochondria. MDA is one of the most important products of membrane lipid peroxidation, and its production can also aggravate membrane damage (Yang et al., 2019). Therefore, the content of MDA can elucidate the degree of membrane lipid peroxidation. Glutathione (GSH) is a tripeptide composed of glutamic acid, cysteine, and glycine, which can maintain normal immune system function and have an antioxidant effect (Shan et al., 2021). SOD is an active protease containing metal elements that can eliminate harmful substances produced by organisms during metabolism. SOD has special physiological activity and is the primary substance for free radical scavenging in organisms (Yang et al., 2019). Notably, our findings showed that the MDA content significantly increased in the mice treated with LPS, and SOD activity and GSH level markedly decreased in serum, while PRP pretreatment could reverse this effect (Figure 4A). Consistently, the results in cardiac tissues were the same (Figure 4B). In addition, we detected the level of ROS in heart tissue, and the immunofluorescence results showed that although the ROS level in mice after LPS treatment was remarkably increased, confirming that PRP pretreatment could distinctly reduce the production of ROS and reduce the damage caused by LPS (Figures 4C,D). Moreover, compared with the control group, the intracellular ROS content in NRCMs in the LPS group was significantly increased, while it was decreased by PRP (Figures 4E,F). These findings provided evidence that PRP could reduce the oxidative stress level induced by LPS and the production of lipid peroxides.

### 3.5 Platelet-rich plasma alleviated ferroptosis induced by lipopolysaccharide

**FIGURE 5:**
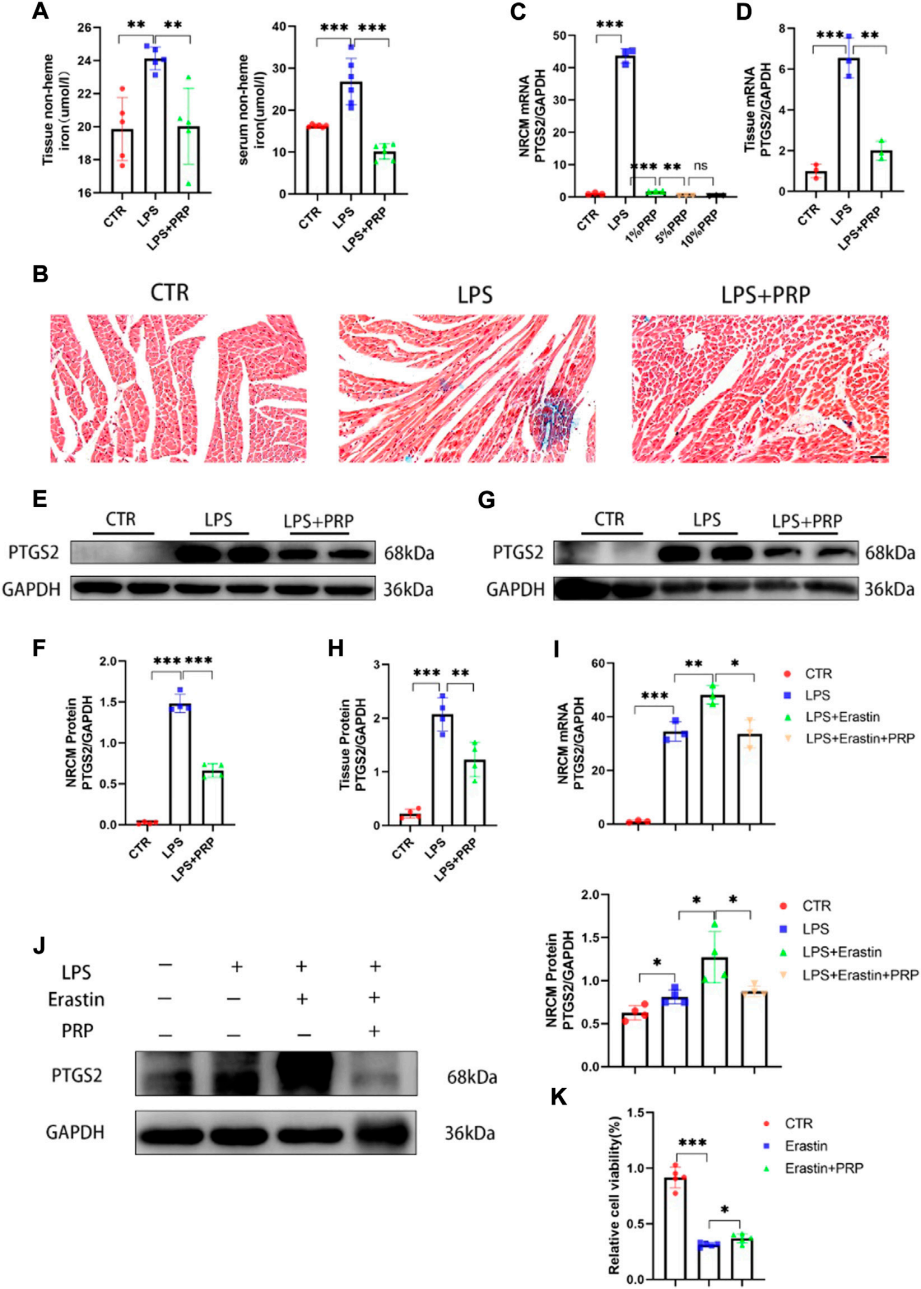
PRP inhibited ferroptosis induced by LPS. **(A)** Iron in tissues and serum were measured after LPS and PRP treatment (*n* = 5, *n* = 6). **(B)** Prussian blue staining for iron in hearts (scale bar = 50 µm). **(C–D)** The mRNA levels of PTGS2 after being treated with PRP in NRCMs and tissues (*n* = 3). **(E–H)** The protein levels of PTGS2 in NRCMs and tissues (*n* = 4). **(I–J)** The mRNA and protein levels of PTGS2 after erastin (10 µM) and PRP treatment for 6 h (*n* = 4). **(K)** Effect of erastin and PRP on cell viability after treated for 24 h (*n* = 5). * indicates *p* < 0.05. ** indicates *p* < 0.01. *** indicates *p* < 0.001.

Ferroptosis is a non-apoptotic form of cell death that depends on intracellular iron accumulation and causes toxic lipid peroxidation and increased ROS. Therefore, we detected the content of non-heme iron in serum and heart tissue to verify the correlation between PRP and ferroptosis. The results showed that compared with the control group, the non-heme iron content in the LPS group was significantly increased, while it was reduced in the LPS + PRP group (Figure 5A). Moreover, the results of Prussian blue staining also confirmed that PRP could reduce the level of ferric iron in heart tissue (Figure 5B). These findings suggested that PRP has a role in regulating iron metabolism. To further confirm the role of PRP in ferroptosis, we detected the expression of PTGS2 *in vivo* and *in vitro*, in addition to ROS, MDA, and GSH levels in mice. NRCM were pretreated with different concentrations of PRP (1%, 5%, and 10%) for 2 h, and then LPS was added for 24 h. The qPCR results demonstrated that PRP could markedly reverse the increase of PTGS2 mRNA content caused by LPS, consistent with the results in tissue (Figures 5C,D). Western blot results also confirmed the above changes (Figures 5E–H). Erastin is an ferroptosis activator that can cause cell death. The qPCR and Western blot results showed that erastin aggravated the ferroptosis induced by LPS, and the content of PTGS2 increased significantly, while the use of PRP alleviated this damage (Figures 5I,J). Then cell viability was evaluated by CCK8 assay. Compared with the control group, erastin treatment resulted in a significant decrease in cell viability which could be rescued by PRP (Figure 5K). These findings demonstrated that PRP played a protective role by inhibiting ferroptosis caused by LPS.

### 3.6 Platelet-rich plasma mitigated the inflammatory process caused by lipopolysaccharide

**FIGURE 6:**
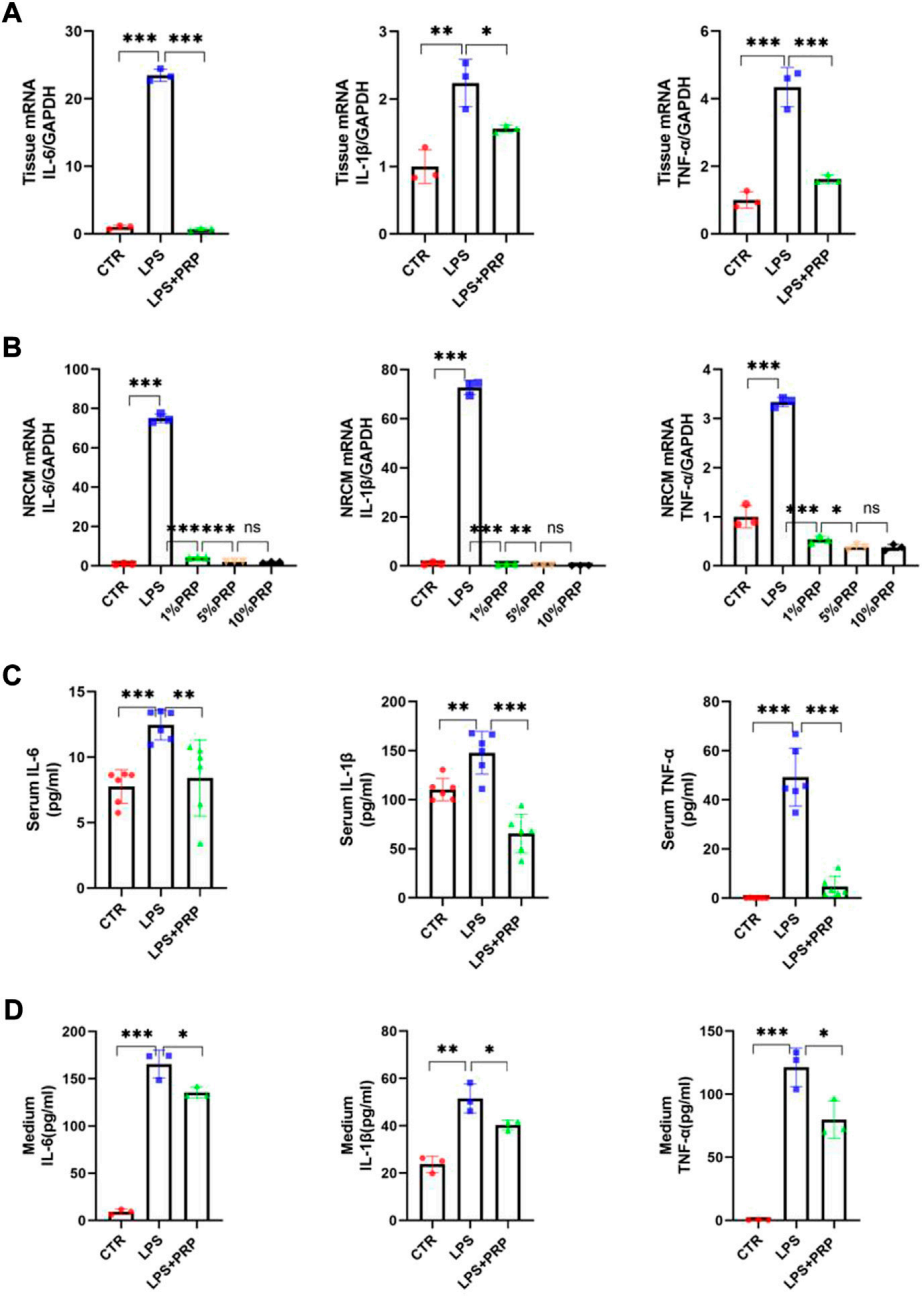
PRP mitigated the inflammation caused by LPS. **(A)** and **(B)** The mRNA expression levels of IL-6, IL-1β, and TNF-α in hearts and NRCMs treated with LPS and PRP (*n* = 3). **(C,D)** The levels of IL-6, IL-1β, and TNF-α in serum and cell culture medium were measured by ELISA (*n* = 6, *n* = 3). * indicates *p* < 0.05. ** indicates *p* < 0.01. *** indicates *p* < 0.001. ns indicates *p* > 0.05.

Growing evidence has confirmed the safety and effectiveness of PRP in controlling inflammation and alleviating injury (Sheean et al., 2021) and that inflammation is inevitable in LPS-induced injury. Therefore, we conducted qPCR and ELISA to verify the role of PRP in LPS-induced inflammation. The qPCR results showed that PRP could significantly reduce the mRNA levels of IL-6, IL-1β, and TNF-α in heart tissue and reverse the inflammatory response induced by LPS (Figure 6A). Consistently, these findings were also observed in NRCM (Figure 6B). Next, we used ELISA kits to detect the levels of inflammatory factors in the serum and cell culture medium. Compared with the LPS group, the content of inflammatory factors in the LPS + PRP group was significantly decreased (Figures 6C,D). These results demonstrated that PRP could regulate the inflammatory signaling pathway activated by LPS.

### 3.7 Platelet-rich plasma played a protective role through the AKT/mTOR signaling pathway

**FIGURE 7:**
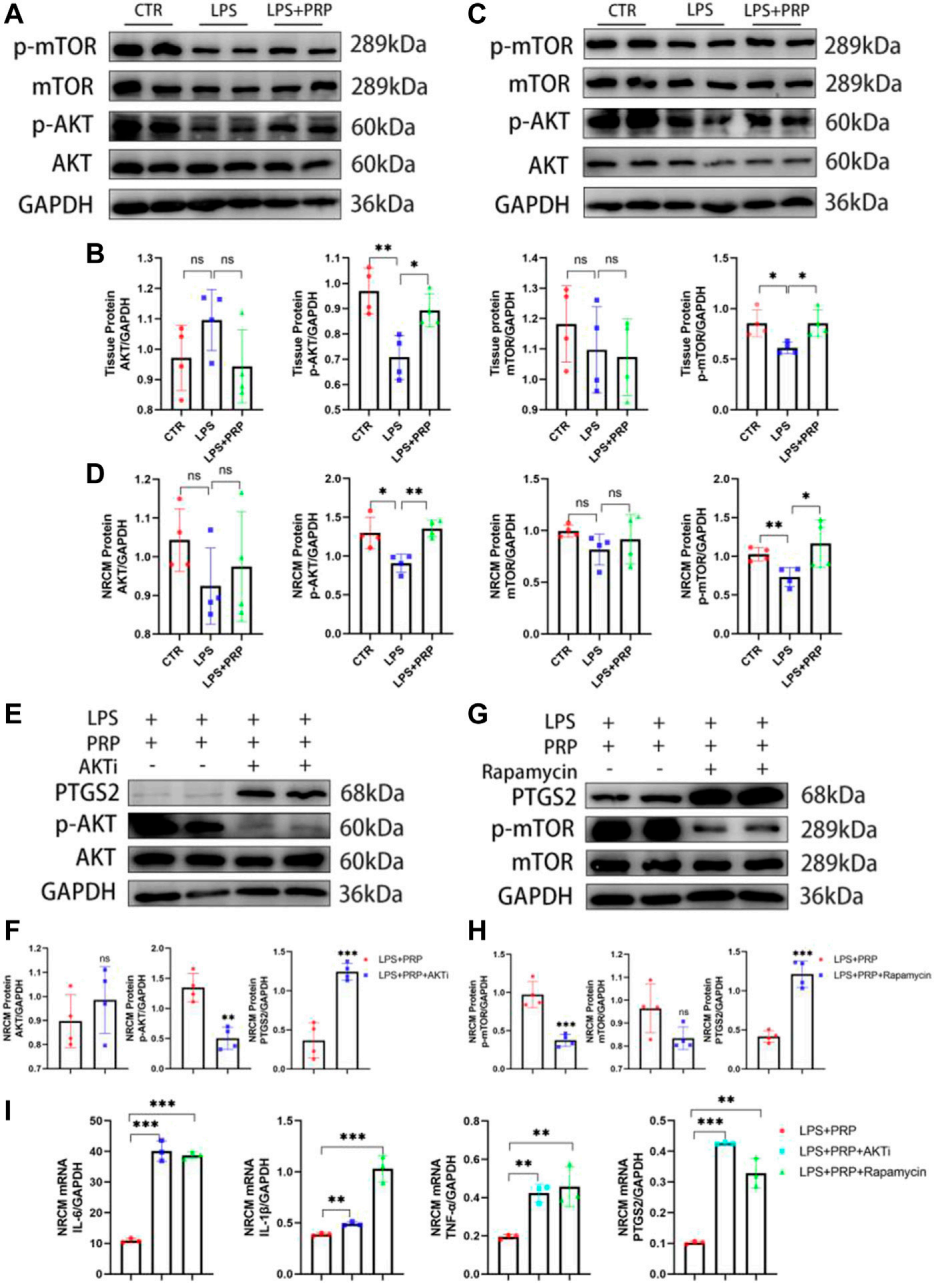
PRP played a protective role through the AKT/mTOR signaling pathway. **(A)** and **(B)** The protein levels of AKT, p-AKT, mTOR, and p-mTOR in heart tissues (*n* = 4). **(C,D)** The protein levels of AKT, p-AKT, mTOR, and p-mTOR in NRCMs (*n* = 4). **(E)** and **(F)** The protein levels of AKT, p-AKT, and PTGS2 in NRCMs after being treated with AKTi (*n* = 4). **(G)** and **(H)** The protein levels of AKT, p-AKT, and PTGS2 in NRCMs after being treated with Rapamycin (*n* = 4). **(I)** The mRNA expression levels of IL-6, IL-1β, TNF-α and PTGS2 in NRCMs treated with AKTi and Rapamycin (*n* = 3). * indicates *p* < 0.05. ** indicates *p* < 0.01. *** indicates *p* < 0.001. ns indicates *p* > 0.05.

The AKT/mTOR signaling pathway plays a critical role in the development of multiple diseases and can regulate various mechanisms such as inflammation, oxidative stress, apoptosis, and autophagy (Jiao et al., 2022; Zhang et al., 2022). At the same time, it has been reported that p-AKT and p-mTOR expressions were down-regulated in cardiomyocytes after treatment with LPS(Qi et al., 2021). Therefore, to verify the role of the AKT/mTOR signaling pathway in LPS-induced cardiac injury, we first performed a Western blot on heart tissues intraperitoneally injected with LPS. The results showed that the expression of p-AKT and p-mTOR in heart tissues and NRCMs was significantly down-regulated after LPS injection, but this effect was significantly reversed after PRP treatment (Figures 7A–D). To further verify the mechanism of action of PRP, we added inhibitors of AKTi and Rapamycin to NRCMs, separately. After pretreatment for 2 h, LPS and PRP were added and the results demonstrated that the protein levels of p-AKT and p-mTOR were decreased, and PTGS2 was increased after being treated with inhibitors (Figures 7E–H). In addition, the qPCR results showed that the protective effect of PRP was diminished after the addition of inhibitors, and the level of inflammation and ferroptosis were once again elevated (Figure 7I). Therefore, we hypothesized that PRP attenuated LPS-induced cardiac injury through the AKT/mTOR signaling pathway.

## 4 Discussion

This study indicated that PRP could play an important protective effect in LPS-induced cardiac injury, which can lower mortality by reducing oxidative stress and ferroptosis levels, inhibiting inflammatory signaling pathways, and improving systolic cardiac function, which is consistent with the previous description of the PRP function. Therefore, PRP could be a new potential treatment target for SIMD.

In recent years, the role of growth factors in wound healing and tissue regeneration has attracted more and more attention, and platelets are currently considered one of the key sources of growth factors (Martínez et al., 2015). Platelet concentrate is a blood derivative product obtained by centrifugation of blood. Due to the high concentration of platelets, it can release growth factors and affect cell proliferation, differentiation, and migration. At present, it has been widely used in many fields such as stomatology, sports medicine, and plastic surgery. According to different preparation methods, it is currently divided into platelet-rich plasma (PRP), platelet-rich fibrin (PRF), and concentrated growth factor (CGF) (Imam et al., 2022). Since PRF and CGF are presented in a gel state, their use in many fields is limited. PRP has a liquid state due to the addition of anticoagulants in the preparation process. Although there are some differences in the preparation process, all of them are essentially concentrated platelet products. A previous study has demonstrated that PRF could significantly promote angiogenesis in the rat myocardial infarction model, especially in the infarction area and edge area, improving myocardial fibrosis and contributing to the recovery of cardiac function (Qian et al., 2022). At the same time, cell studies have found that PRP could stabilize the mitochondria and reduce ROS production in ischemia-reperfusion hearts (Hargrave and Li, 2012).

In human clinical trials, PRP is only used to treat chronic diseases with continuous inflammatory processes, manifested in the imbalance of anti-inflammatory and proinflammatory factors, the insufficient scavenging capacity of reactive oxygen species, and excessive production. Growth factors and cytokines have been confirmed to improve the above pathological process. In patients with diabetic foot ulcers, local application of PRP alleviated pain and inflammatory response and promoted granulation tissue formation and wound healing (Babaei et al., 2017). Based on the above research, we speculated that PRP could also play a critical role in SIMD. The results showed that after treatment with LPS, cardiac contractility was impaired, which was manifested as decreased LVEF and FS, and increased LDH levels in serum. However, PRP significantly reversed this adverse reaction, improved heart function, and reduced mortality.

It is currently believed that inflammation plays a key role in SIMD. The results showed that LPS could activate the inflammatory signaling pathway *in vivo* and induce myocardial cells and macrophages to release many inflammatory factors, such as IL-6, IL-1β, and TNF-α. Many inflammatory factors would further interfere with energy metabolism and calcium homeostasis, resulting in systolic cardiac dysfunction, reduced ejection fraction, multiple organ dysfunction syndromes (MODS), and ultimately lead to death (Szekely and Arbel, 2018). Therefore, blocking the inflammatory response process is of great significance for the treatment of SIMD. There is considerable evidence that PRP plays an important role in alleviating inflammatory injury, promoting tissue repair, and wound healing. Researchers found that local injection of PRP into the articular cavity could significantly reduce the apoptosis of chondrocytes induced by adriamycin and reduce the level of inflammatory markers by blocking the NF-κB signaling pathway, thereby improving the degree of cartilage damage (Zhu et al., 2020). In addition, other studies have shown that PRP could promote the recovery of damaged muscles, improve myocardial injury in diabetes, and accelerate wound healing, and its mechanism is related to the regulation of inflammatory response (Tsai et al., 2018; Xu P. et al., 2020; Abdel Hafez et al., 2021). In this study, our findings confirmed the role of PRP in the inflammatory response of mice and NRCM. In addition, this study showed for the first time that PRP could inhibit the myocardial proinflammatory signaling pathway and reduce the levels of inflammatory mediators such as IL-6, IL-1β, and TNF-α in LPS-induced cardiac injury. Although it is generally believed that controlling inflammatory response can improve the adverse effects of LPS, some studies argue against it. Researchers have found that even specific treatments for inflammatory pathways, such as blocking IL-1β or anti-TNF-α treatment, still cannot reduce the mortality of patients (Drosatos et al., 2015; Szekely and Arbel, 2018), which implies that controlling the inflammatory signaling pathway alone is insufficient, and multiple mechanisms must be considered.

Ferroptosis is a regulated form of cell death characterized by the iron-dependent accumulation of lipid peroxidation and will seriously affect the state of cells, mainly manifested as mitochondrial changes. Excessive iron production will change the fluidity and integrity of the plasma membrane, resulting in mitochondrial shrinkage, cristae reduction or even disappearance, and ultimately affect the function of mitochondria, but the morphology of the nucleus did not change significantly (Li J. et al., 2020). Ferroptosis can be triggered by external or internal pathways. The external pathway is initiated by regulating transporters, while the internal pathway mainly blocks the expression or activity of intracellular antioxidant enzymes so that the oxidation and antioxidant system of cells are unbalanced. As a result, glutathione (GSH) depletion and glutathione peroxidase 4 (GPX4) activity in cells are reduced, and lipid peroxides are not decomposed in time. Through Fenton reaction and other peroxidation, a large number of reactive oxygen species are generated under the catalysis of ferrous ions, leading to lipid oxidation on cell membranes. The accumulation of toxic lipid peroxides, in turn, promotes the generation of reactive oxygen species, induces further peroxidation of cell membrane lipids, causes selective cell membrane permeability loss, and destroys its integrity (Qiu et al., 2020). In addition, stress-related factors such as temperature, hypoxia, or radiation can also induce cell ferroptosis (Tang and Kroemer, 2020). In addition, the regulation of Nrf2 signaling pathway and MAPK signaling pathway can improve the progression of ferroptosis and reduce injury (Li Y. et al., 2020; Xu et al., 2021).

At present, it is believed that ferroptosis is related to the occurrence and development of various diseases, including tumors, Parkinson’s disease, cerebral hemorrhage, etc. In recent years, more studies have focused on the relationship between ferroptosis and cardiovascular diseases. Ferroptosis is involved in the pathogenesis of atherosclerosis through inflammation, endothelial dysfunction, and foam cell formation, while ferroptosis inhibitors reverse these effects (Luo et al., 2021). During ischemia/reperfusion injury, there was no significant change in the level of ferroptosis biomarkers at a different time during ischemia. However, with the prolongation of reperfusion time, the GPX4 level in myocardial tissue decreased, and iron concentration gradually increased, indicating that ferroptosis mainly occurred during reperfusion (Lillo-Moya et al., 2021). If a relevant intervention were carried out in time, it would help to reduce the infarct size. In addition, LI et al. (Li W. et al., 2019) found that ferrostatin-1 can inhibit the ferroptosis of fibroblasts and the adhesion of neutrophils to coronary artery endothelial cells in ischemia-reperfusion injury induced by heart transplantation, indicating that intervention with ferroptosis inhibitors before heart transplantation could improve the prognosis of patients.

Although the specific mechanism remains to be further studied, ferroptosis has been found to play an important role in cardiovascular diseases such as cardiomyopathy, myocardial infarction, ischemia/reperfusion injury, and heart failure. Selecting ferroptosis as a target for regulation could provide an effective treatment for cardiovascular diseases (Li et al., 2021). In this study, we proved for the first time that PRP alleviated the development of SIMD by inhibiting the ferroptosis signaling pathway. First, we detected the levels of oxidative stress and lipid peroxidation in mice. Although the injection of LPS could decrease SOD activity and GSH level and increase MDA content, PRP treatment reversed this undesirable phenomenon. The immunofluorescence results of ROS in mouse tissues also confirmed this conclusion. Fang et al. (Fang et al., 2019) used adriamycin to establish a heart injury model. It was found that the release of free iron increased with the degradation of heme led to ferroptosis in myocardial cells and eventually heart failure. Ferroptosis inhibitor treatment could play a significant protective effect, reducing heart injury and heart failure and demonstrating that the iron content in the body affects the progress of ferroptosis. Therefore, we next detected the iron content in mice and further confirmed that PRP inhibited the release of non-heme iron *in vivo* and reduced ferroptosis. Finally, we verified the PTGS2 content at the gene and protein levels. As the most important rate-limiting enzyme in arachidonic acid metabolism, PTGS2 is closely related to ferroptosis (Li N. et al., 2020). It was found that the content of PTGS2 was significantly increased after LPS treatment, consistent with our results, while the expression could be significantly reduced by using PRP, which further confirmed the correlation between PRP and ferroptosis.

**FIGURE 8:**
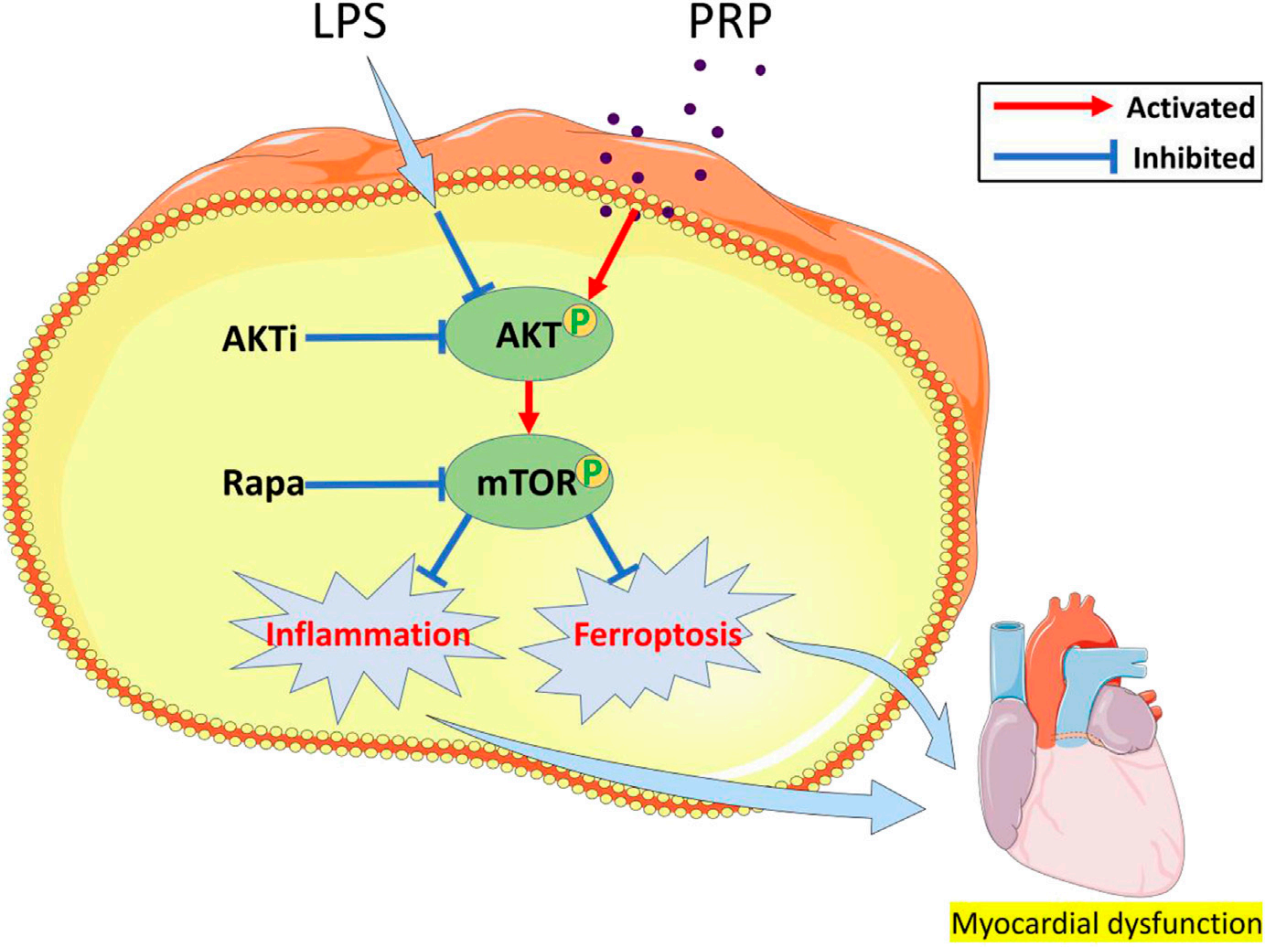
A schematic diagram of the effect of PRP on LPS-induced cardiac injury through the AKT/mTOR pathway.

Next, we explored the mechanisms of PRP in septic cardiomyopathy. Previous studies have shown that LPS activates NF-kB mediated inflammatory signaling pathway, inhibition of this signaling pathway can attenuate LPS-induced myocardial injury (Xu X. et al., 2020). In addition, the regulation of MAPK and JNK/ERK signaling pathways also plays a protective role (Ning et al., 2021; Zhu et al., 2022). Recently, it was found that Neferine can alleviate LPS-induced oxidative stress through AKT/mTOR signaling pathway (Qi et al., 2021), and it was reported that ferroptosis could be attenuated by activating AKT/mTOR signaling pathway (Cheng et al., 2022), which provides a new idea for our study. The AKT/mTOR signaling pathway is involved in the development of multiple diseases. It was found that PRP can significantly affect the expression of AKT and mTOR, and our study confirmed that the expression of p-AKT and p-mTOR was significantly elevated after treatment with PRP, further establishing that PRP can activate the AKT/mTOR pathway. Therefore, we used the pathway inhibitors, Akti and Rapamycin, to confirm that PRP attenuates inflammation and ferroptosis through the AKT/mTOR signaling pathway. The results showed that Akti and Rapamycin reversed the protective effect of PRP, and the inflammation level and protein expression of ferroptosis were significantly increased. These findings indicate that the AKT/mTOR pathway is connected to the protective role of PRP in ameliorating LPS-induced cardiac injury (Figure 8).

## 5 Conclusion

This study showed that PRP improved LPS-induced oxidative stress and ferroptosis levels and reduced the inflammatory response *via* the AKT/mTOR signaling pathway. Our findings provide a basis for PRP as a potential treatment for septic cardiomyopathy.

## Data Availability

The original contributions presented in the study are included in the article, further inquiries can be directed to the corresponding authors.
